# Author Correction: Truly privacy-preserving federated analytics for precision medicine with multiparty homomorphic encryption

**DOI:** 10.1038/s41467-021-26885-6

**Published:** 2021-11-11

**Authors:** David Froelicher, Juan R. Troncoso-Pastoriza, Jean Louis Raisaro, Michel A. Cuendet, Joao Sa Sousa, Hyunghoon Cho, Bonnie Berger, Jacques Fellay, Jean-Pierre Hubaux

**Affiliations:** 1 Laboratory for Data Security, EPFL, Lausanne, Switzerland; 2 Precision Medicine Unit, Lausanne University Hospital, Lausanne, Switzerland; 3 Data Science Group, Lausanne University Hospital, Lausanne, Switzerland; 4 Precision Oncology Center, Lausanne University Hospital, Lausanne, Switzerland; 5 Broad Institute of MIT and Harvard, Cambridge, MA USA; 6 Computer Science and AI Laboratory, MIT, Cambridge, MA USA; 7 Department of Mathematics, MIT, Cambridge, MA USA; 8 School of Life Sciences, EPFL, Lausanne, Switzerland

**Keywords:** Computational biology and bioinformatics, Machine learning, Medical research

Correction to: *Nature Communications* 10.1038/s41467-021-25972-y, published online 11 October 2021.

The original version of this Article omitted a reference to previous work in ‘Blatt, M., Gusev, A., Polyakov, Y. & Goldwasser, S. Secure large-scale genome-wide association studies using homomorphic encryption. *Proc. Natl. Acad. Sci. USA* **117**, 11608–11613 (2020)’. This has been added as reference 25 at the fifth paragraph of the Introduction, second line: ‘More sophisticated solutions for FA, which aim to provide end-to-end privacy protection, including for the shared intermediate data, have been proposed^15–25^.’. This has been corrected in the PDF and HTML versions of the Article.

