# Supplementary material for: Truly privacy-preserving federated analytics for precision medicine with multiparty homomorphic encryption
Source: Nat Commun. 2021 Oct 11;12:5910. doi: 10.1038/s41467-021-25972-y (PMC8505638; doi:10.1038/s41467-021-25972-y)
Supplement: Supplementary file 1 — Supplementary Information [file 41467_2021_25972_MOESM1_ESM.pdf]

Supplementary Notes

Truly Privacy-Preserving Federated Analytics for Precision Medicine with Multiparty Homomorphic Encryption

David Froelicher[1]
Juan R. Troncoso-Pastoriza[1]
Jean Louis Raisaro[2,3]
Michel A. Cuendet[4]
Joao Sa Sousa[1]
Hyunghoon Cho[5]
Bonnie Berger[5,6,7]
Jacques Fellay[2,8]
Jean-Pierre Hubaux[1,*]

[1] Laboratory for Data Security, EPFL, Lausanne, Switzerland
[2] Precision Medicine Unit, Lausanne University Hospital, Lausanne, Switzerland
[3] Data Science Group, Lausanne University Hospital, Lausanne, Switzerland
[4] Precision Oncology Center, Lausanne University Hospital, Lausanne, Switzerland
[5] Broad Institute of MIT and Harvard, Cambridge, Massachusetts, USA
[6] Computer Science and AI Laboratory, MIT, Cambridge, Massachusetts, USA
[7] Department of Mathematics, MIT, Cambridge, Massachusetts, USA
[8] School of Life Sciences, EPFL, Lausanne, Switzerland
*_jean-pierre.hubaux@epfl.ch_

Supplementary Note 1: Cryptographic Background

In FAMHE, the data exchanged by the data providers are always encrypted such that only the querier can decrypt the final result. For this purpose, we rely on a multiparty (or distributed) fully-homomorphic encryption scheme[1] in which the secret key is distributed among the parties and the corresponding collective public key pk is publicly known. Thus, each party can independently compute on ciphertexts encrypted under pk, but all parties have to collaborate to decrypt a ciphertext. Hence, as long as one data provider is honest and refuses to participate in the decryption, encrypted data cannot be decrypted. This multiparty scheme also enables DPs to collectively switch the encryption key of a ciphertext from pk to another public key, i.e., the querier's key, without decrypting. We provide a list of recurrent symbols in Supplementary Table 3. Mouchet et al.[1] propose a multiparty version of the Brakerski Fan-Vercauteren (DFV) lattice-based homomorphic cryptosystem[2] and introduce interactive protocols for key management and cryptographic operations. We rely directly on this multiparty scheme for the computation of Kaplan-Meier survival curves, which involves only exact integer arithmetic, and we use an adaptation to the Cheon-Kim-Kim-Song cryptosystem (CKKS)[3] (described by Froelicher et al.[4]) that enables approximate arithmetic for the GWAS operations. Froelicher et al.[4] showed that this adaptation satisfies similar security properties to the original scheme proposed by Mouchet et al.[1]. The security comes mainly from the fact that the underlying (centralized) cryptoschemes, i.e., BFV and CKKS, share the same computational assumptions and are based on the same hard problem, i.e., the decisional RLWE problem[5]. In Optimization Techniques (Methods), we discuss the SIMD property of these cryptosystems and how FAMHE builds on it to efficiently execute FA workflows with encrypted data.

Supplementary Note 2: Related Work

Centralized solutions for medical-data sharing[6–8] require large amounts of data to be stored in a single repository that becomes a single point of failure and that (often) has to be fully trusted.

To alleviate this trust assumption, federated-learning solutions[9–11] were proposed. In these solutions, the data providers keep their data locally and share only aggregates or training-model updates with a central server. However, multiple research contributions[12–14] have shown that these aggregates can still reveal significant information about the data providers' data. For example, Nasirigerdeh et al. proposed sPLINK[10], a federated instantiation of the PLINK[15] software to perform a GWAS. With sPLINK the data providers' partial covariance matrices (i.e., intermediate result) are revealed to the server that aggregates these matrices in order to perform the models training. Although the original data $\mathbf{X}$ is not actually transferred, some information about the original data can be inferred from the covariance matrix $\mathbf{X}^{\mathbf{T}}\mathbf{X}$ computed by the aggregating server. In FAMHE, the covariance matrix is collectively and obliviously computed by exchanging encrypted data such that the models can be trained without revealing any intermediate data.

Similarly, secure multiparty solutions[16,17] rely on secret-sharing to compute on medical data without revealing intermediate or aggregate information. Cho et al.[17] designed a three-party secret-sharing-based solution for enabling GWAS execution while not revealing information on the input data. Secret-sharing-based solutions require the data providers to communicate their data to a limited number of computing nodes, i.e., outside their premises. FAMHE efficiently scales to federated learning settings where many DPs locally keep their data.

Distributed solutions relying on homomorphic encryption[18–21] to enable federated analytics in a trust model similar to FAMHE were proposed. Some of these works assume a threat model more constraining than FAMHE, as they consider an active malicious adversary, but also exclusively focus on simple computations, e.g., counts and simple statistics. To propose a generic federated workflow for biomedical federated analytics, we build on the multiparty homomorphic encryption-based protocols proposed by Froelicher et al.[4]. We show how the sophisticated GWAS computation can be efficiently performed through this workflow.

Differential-privacy-based solutions[22–24], in which the intermediate values are obfuscated by a specific amount of noise, assume a paradigm different than FAMHE, as privacy is traded off with accuracy. In fact, this obfuscation decreases the data and model utility. The training of accurate models requires high-privacy budgets, but the achieved privacy level remains unclear[25]. In FAMHE, similarly to standard cleartext non-secure solutions (e.g., PLINK[15]), the accuracy is traded for only the performance. We show in Results that FAMHE achieves an accuracy similar to standard non-secure solutions, and that it is able to scale to a high number of data providers and yields an acceptable execution time.

In Table 1, we compare certain existing solutions and FAMHE on multiple criterion: whether (i) patient levels and (ii) aggregated data are protected, (iii) the data protection satisfies the GDPR definition of anonymity, (iv) the solutions scale with the number of data providers and computing parties, (v) a majority of computing parties can be dishonest (without deviating from the protocol), (vi) the obtained results are the same as if they were computed on a centralized cleartext dataset (i.e., utility), (vii) the system enables multiple types of computations, and (viii) the solution is tested in a real application scenario. A "$\sim$" means that the property is partially fulfilled.

	Protect. Mecha.	Patient Level Data Protect.	GDPR Anonym.	Intermed. Val. Protect.	Scale w. Parties	Passive Dishonest Majority	Preserve Utility	Comput. Flexibility	Complex Comput.	Appl. To Real Cases
AllOfUs[6] Gen. Eng.[7] UKBio.[8]	None	No	No	No	~	No	Yes	Yes	Yes	Yes
DataSHIELD[11] Vantage6[26] SHRINE[27] Splink[10] SWARM[28]	Aggregates	~	No	No	Yes	No	~	~	Yes	~
Bonomi et al.[22]	DiffP	Yes	No	~	Yes	Yes	No	No	No	~
Li et al.[23]	DiffP	Yes	No	~	Yes	Yes	No	No	Yes	No
Cho et al.[17]	SMC	Yes	Yes	Yes	No	Yes	~	No	Yes	Yes
Jagadeesh et al. [16]	SMC	Yes	Yes	Yes	No	No	~	No	No	Yes
Froelicher et al.[20] Lu et al.[21]	MHE	Yes	Yes	Yes	Yes	Yes	~	~	No	No
**FAMHE**	MHE	Yes	Yes	Yes	Yes	Yes	~	~	Yes	Yes

Supplementary Table 1. Comparison of Existing Solutions for biomedical FA.

Supplementary Note 3: Secure & Distributed Computation of a Kaplan-Meier Survival Curve

Supplementary Figure 1 depicts FAMHE secure and federated workflow for the computation of a survival curve (see Methods). Each DP_i (with $i = 0,\ldots,S$) locally computes, encodes and encrypts a vector of the form $n_0^{(i)}, c_0^{(i)}, d_0^{(i)}, \ldots, n_T^{(i)}, c_T^{(i)}, d_T^{(i)}$ containing the values $n_j^{(i)}$ (number of survivors), $c_j^{(i)}$ (number of censored), $d_j^{(i)}$ (number of deceased) corresponding to each time point t_j for $t_j = 0,\ldots,T$. The DPs' vectors are then collectively aggregated and the final result is collectively switched from the public key pk to the querier's public key, who can decrypt the result with its secret key and generate the curve.

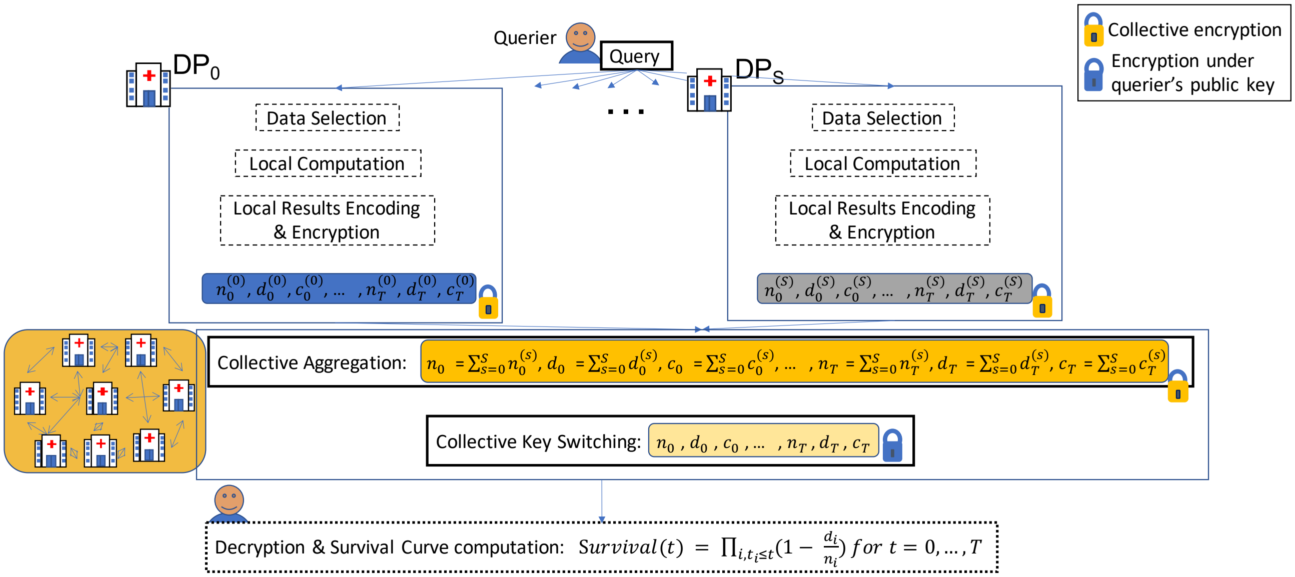

Supplementary Figure 1. Secure & Distributed Computation of a Survival Curve.

Supplementary Note 4: Secure & Distributed Computation of a GWAS

Supplementary Figure 2 depicts FAMHE secure and federated workflow for the computation of a Genome-Wide Association Study (see Methods). In Supplementary Figure 2a, we describe FAMHE's protocol to compute an exact GWAS. First the covariance matrix ($\mathbf{X}^{\mathbf{T}}\mathbf{X}$) is collectively computed (Collective Aggregation, CA) by the DPs before being inversed in the encrypted domain by one data provider (DP_R) in Step 1. In Step 2, the inversed matrix is augmented with the variant's contribution (vector $\mathbf{u}$ that contains the variant's value for each patient) by following the Shermann-Morrison formula[29] and the method presented in the report on Cryptographic and Privacy-preserving primitives (page 52) of the WITDOM European project[30]. We remark that whenever possible, the DPs locally compute on their cleartext data before securely aggregating their partial results. The matrix $\mathbf{W}$ is made of 3 submatrices ($\mathbf{W_{11}}$, $\mathbf{W_{12}}$,$\mathbf{W_{21}}$) that are computed in Step 2. In Step 3, the model weights (or coefficients, $\mathbf{w}$) are computed such that all elements required to obtain the p-value corresponding to the variant's coefficient can be computed in Step 4. In Step 5, the results are switched (Key Switching, KS) to the querier's public key such that they can compute the final result in Step 6. We remark that this process is performed simultaneously on multiple variants thanks to the SIMD property of the cryptoscheme, as described in Supplementary Figure 3. In Protocol 2b, we show how the GWAS can be estimated and therefore computed with a lower complexity by avoiding the computation of the complete inverse matrix of the covariance matrix. The steps are similar as in Protocol 2a except that in Step 2, the model coefficients corresponding to the covariates are obtained through an efficient stochastic gradient descent with the label (phenotype) $\mathbf{y}$. We rely on the protocols proposed by Froelicher et al.[4] to perform the SGD on an encrypted model. Step 3 corresponds to a partial execution of steps 2 and 3 from Protocol 2a. In Step 4, we compute the coefficient corresponding to the variant and the other elements required to obtain the p-value.

a FAMHE-GWAS.

Step 1: Inverse of covariance matrix of covariates

Each DP$_i$: $\mathbf{X}_i = [\mathbf{1}, \mathbf{X}_i]$ with $\mathbf{X}_i \in \mathbb{R}^{(p_i \times (f+1))}$

Each DP$_i$ computes $\mathbf{X}_i^T \mathbf{X}_i$ with $\mathbf{X}_i^T \mathbf{X}_i \in \mathbb{R}^{((f+1)\times(f+1))}$

CA $\rightarrow$ E($\mathbf{X}^T\mathbf{X}$)

DP$_R$: E$((\mathbf{X}^T\mathbf{X})^{(-1)})$ = GJ(E$((\mathbf{X}^T\mathbf{X})$)) and broadcasts

Step 2: Variants contribution

For each column $\mathbf{u}$ in $\mathbf{V}$ do:

Each DP$_i$ computes $\mathbf{u}_i^T\mathbf{u}_i$, $\mathbf{u}_i^T\mathbf{X}_i \times$ E$((\mathbf{X}^T\mathbf{X})^{(-1)})$

CA $\rightarrow$ E($\mathbf{u}^T\mathbf{u}$), E($\mathbf{W}_{21}$) = E($\mathbf{u}^T\mathbf{X}(\mathbf{X}^T\mathbf{X})^{(-1)}$))

DP$_R$ broadcasts E($\mathbf{u}^T\mathbf{X}(\mathbf{X}^T\mathbf{X})^{(-1)}$))

Each DP$_i$ computes E($\mathbf{u}^T\mathbf{X}(\mathbf{X}^T\mathbf{X})^{(-1)}$)) $\times \mathbf{X}_i^T\mathbf{u}_i$

CA $\rightarrow$ E($\mathbf{u}^T\mathbf{X}(\mathbf{X}^T\mathbf{X})^{(-1)}\mathbf{X}^T\mathbf{u}$)

DP$_R$ computes $\mathbf{E}(\frac{1}{c})$ = E($\mathbf{u}^T\mathbf{u} - \mathbf{u}^T\mathbf{X}(\mathbf{X}^T\mathbf{X})^{(-1)}\mathbf{X}^T\mathbf{u}$)

Each DP$_i$ computes E$((\mathbf{X}^T\mathbf{X})^{(-1)})$) $\times \mathbf{X}_i^T\mathbf{u}_i$

CA $\rightarrow$ E($\mathbf{W}_{12}$) = E$((\mathbf{X}^T\mathbf{X})^{(-1)}\mathbf{X}^T\mathbf{u})$

DP$_R$ computes E($\mathbf{W}_{11}$)=E($\frac{1}{c}(\mathbf{X}^T\mathbf{X})^{(-1)} + \mathbf{W}_{12}\mathbf{W}_{21}$)

E($\mathbf{W}$) = E$\left(\begin{smallmatrix}\mathbf{W}_{11} & \mathbf{W}_{12} \\ \mathbf{W}_{21} & 1\end{smallmatrix}\right)$

Step 3: All coefficients

Each DP$_i$ computes $[\mathbf{X}_i, \mathbf{u}_i]^T\mathbf{y}_i$

CA $\rightarrow$ E(w) = E($\mathbf{W} \times [\mathbf{X}, \mathbf{u}]^T\mathbf{y}$)

Step 4: P-value elements

Each DP$_i$: E($\mathbf{y}_i'$) = $\mathbf{X}_i \times$ E($\mathbf{w}$) and E(mse$_i$) = $\frac{1}{p}\sum_{j=0}^{p_i}$(E($\mathbf{y}_i'[j]$) $- \mathbf{y}_i[j])^2$

CA $\rightarrow$ E(mse)

Step 5: Key Switching

KS$_Q$(E(mse),E($\frac{1}{c}$,E($\mathbf{w}[f+2]$])) and send to Q

Step 6: Querier final result

Querier decrypts and $p_{\text{val}} = 2 \cdot \text{pnorm}(-|\frac{\mathbf{w}[f+2]}{\sqrt{(\text{mse}\cdot c)}}|)$

b FAMHE-FastGWAS.

Step 1: Inverse of covariance matrix of covariates

Each DP$_i$: $\mathbf{X}_i = [\mathbf{1}, \mathbf{X}_i]$ with $\mathbf{X}_i \in \mathbb{R}^{(p_i \times (f+1))}$

Each DP$_i$ computes $\mathbf{X}_i^T \mathbf{X}_i$ with $\mathbf{X}_i^T \mathbf{X}_i \in \mathbb{R}^{((f+1)\times(f+1))}$

CA $\rightarrow$ E($\mathbf{X}^T\mathbf{X}$)

DP$_R$: E$((\mathbf{X}^T\mathbf{X})^{(-1)})$ = GJ(E$((\mathbf{X}^T\mathbf{X})$)) and broadcasts

Step 2: Covariates coefficients & error

E($\mathbf{w}[1:f+1]$) = SGD($\mathbf{X}, y$) and broadcasts

Each DP$_i$: E($\mathbf{y}_i''$) = $\mathbf{y}_i -$ E($\mathbf{X}_i\mathbf{w}[1:f+1]$)

Each DP$_i$: E($\bar{\mathbf{y}_i''}$) = $\frac{1}{p}\sum_{j=0}^{p_i} E(\mathbf{y}_i''[j])$

Step 3: Variants contribution

For each: column $\mathbf{u}$ in $\mathbf{V}$ do:

Each DP$_i$ computes $\mathbf{u}_i^T\mathbf{u}_i$, $\mathbf{u}_i^T\mathbf{X}_i \times$ E$((\mathbf{X}^T\mathbf{X})^{(-1)})$

CA $\rightarrow$ E($\mathbf{u}^T\mathbf{u}$), E($\mathbf{W}_{21}$) = E($\mathbf{u}^T\mathbf{X}(\mathbf{X}^T\mathbf{X})^{(-1)}$))

DP$_R$ broadcasts E($\mathbf{u}^T\mathbf{X}(\mathbf{X}^T\mathbf{X})^{(-1)}$))

Each DP$_i$ computes E($\mathbf{u}^T\mathbf{X}(\mathbf{X}^T\mathbf{X})^{(-1)}$)) $\times \mathbf{X}_i^T\mathbf{u}_i$

CA $\rightarrow$ E($\mathbf{u}^T\mathbf{X}(\mathbf{X}^T\mathbf{X})^{(-1)}\mathbf{X}^T\mathbf{u}$)

Step 4: P-value elements

DP$_R$ compute E($\frac{1}{c}$) = E($\mathbf{u}^T\mathbf{u} - \mathbf{u}^T\mathbf{X}(\mathbf{X}^T\mathbf{X})^{(-1)}\mathbf{X}^T\mathbf{u}$)

Each DP$_i$: E($\bar{u}_i$) = $\frac{1}{p}\sum_{j=0}^{p_i}$E($\mathbf{u}_i[j]$)

CA $\rightarrow$ E($\bar{\mathbf{y}''}$), E($\bar{u}$) and broadcasts

Each DP$_i$: E(d_i) = $\sum_{j=0}^{p_i}(\mathbf{u}_i[j] -$ E($\bar{\mathbf{u}}$))^2 and E(t_i) = $\sum_{j=0}^{p_i}(\mathbf{u}_i[j] -$ E($\bar{\mathbf{u}}$))($\mathbf{y}_i''[j] -$ E($\bar{\mathbf{y}''}$))

CA $\rightarrow$ E(d), E(t) and broadcasts

Each DP$_i$: E(mse$_i'$) = $\sum_{j=0}^{p_i}$(E(d)$\mathbf{y}_i''[j] -$ E(t)$\mathbf{u}[j])^2$

CA $\rightarrow$ E(mse$'$)

Step 5: Key Switching

KS$_Q$(E(mse$'$),E($\frac{1}{c}$),E(t),E(d) and send to Q

Step 6: Querier final result

Querier decrypts and $p_{\text{val}} = 2 \cdot \text{pnorm}(-|\frac{\mathbf{t}/\mathbf{d}}{\sqrt{\frac{1}{\mathbf{d}^2}(\text{mse}'\cdot c)}}|)$

Supplementary Figure 2. Secure & Distributed Computation of a GWAS. CA stands for collective aggregation and KS for collective key switching.

In Supplementary Figure 3, we describe how the values are packed in order to perform the computations of the main variables used in the protocols presented in Supplementary Figure 2. We note that 13 covariates are considered in the study that we reproduced[31]. We provide here the main intuition behind the packing of each element (in Supplementary Figure 3) and its high level cost in terms of operations:

1. The inverse computation (line 4 in Protocol 2b & 2a) requires row operations on the input matrix. The matrix is therefore row-wise encrypted and its rows are duplicated to enable an efficient use of SIMD and to perform the subsequent operations for multiple variants simultaneously, thus minimizing rotations on encrypted data.

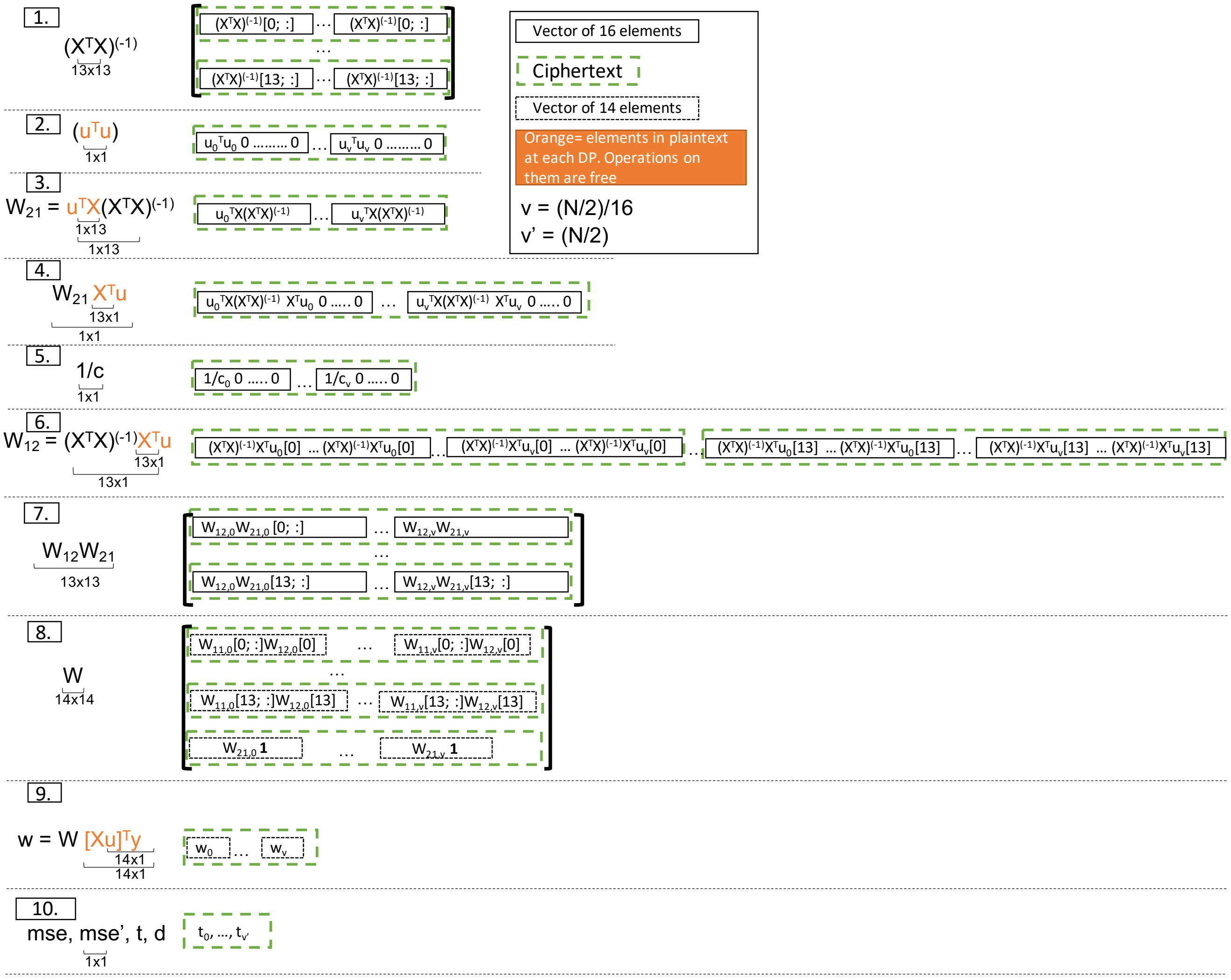

Supplementary Figure 3. Optimized Packing for a Simultaneous Computation on v Variants.

2. The vectors multiplication results are packed such that they are in the right positions to perform the subtraction in line 11 for Protocol 2a and line 14 for Protocol 2b. This packing can be performed by the data providers on their cleartext data and does not require rotations of encrypted ciphertexts.

3. Each element of the plaintext $\mathbf{u}^T\mathbf{X}$ is replicated to be multiplied with a row of $(\mathbf{X}^T\mathbf{X})^{(-1)}$ and the 13 multiplication results are aggregated. This operation does not require any rotation either.

4. The dot product between the plaintext vector $\mathbf{X}^T\mathbf{u}$ and each encrypted row of $\mathbf{W}_{21}$ is performed and the result is packed such that it can be used as such in the subsequent subtraction (line 11 for Protocol 2a and line 14 for Protocol 2b). The dot product requires $\log_2(\text{vectorsize})$ rotations. We note here that all vectors are padded with zeros such that their size is a power of 2. This is done to optimize the number of rotations required.

5. As before, the scalar values for v variants are packed to simplify the subsequent subtraction.
6. As in 4, the dot product is performed between the plaintext vector $\mathbf{X}^T\mathbf{u}$ and each encrypted row of the matrix $(\mathbf{X}^T\mathbf{X})^{(-1)}$. The results are then duplicated to prepare the multiplication of line 14 in Protocol 2a. This operation requires $2 \times \log_2(\text{vectorsize})$ rotations.
7. Each of the 13 ciphertexts of $\mathbf{W}_{12}$ is multiplied with $\mathbf{W}_{21}$. This operation does not require any rotation as the values have been prepared (packed) for this multiplication beforehand (in 6).
8. To construct the matrix W for v variants, we include $\mathbf{W}_{12}$ directly in the padding of W_{11}. Due to $\mathbf{W}_{12}$ packing, this is done in one mask (multiplication with binary vector) and one addition and does not require any rotation. Similarly, one addition is performed to include the 1 values in $\mathbf{W}_{21}$.
9. The dot product between each encrypted row of W and $[\mathbf{Xu}]^T\mathbf{y}$ is performed such that the result can be packed to prepare the multiplication of line 17 in Protocol 2a. This requires $\log_2(\text{vector/rowsize}) + (\text{vector/row} - 1)$ rotations.
10. All elements are computed for v variants simultaneously.

Supplementary Note 5: Quantitative Comparison of FAMHE with existing approaches.

FAMHE is specifically designed to benefit from its multiparty construction and to optimize its use of MHE to efficiently execute secure FA workflows among a large number of data providers that keep their data locally. A non-secure centralized solution based on PLINK takes 14 seconds when performed by a single data provider on the pooled dataset. FAMHE efficiently distributes its workload and achieves an execution time of 69 minutes when the data are split among 12 DPs for the same computation. A non-secure federated solution based on PLINK's meta-analysis method takes around 5 minutes when executed on 12 DPs but yields very imprecise results. We remark that in the meta-analysis the DPs exchange information only once at the end and a non-secure solution in which the DPs collaborate during the process would be slightly slower, e.g., between 5 and 10 minutes, depending on the communication settings. Differential-privacy-based solutions usually yield the same execution time as non-secure solutions. We estimate that a centralized HE-based solution, i.e., executing FAMHE-GWAS and FAMHE-FastGWAS in a centralized manner, would take at least 2228 (with the FastGWAS approach) and 30,781 minutes (with the GWAS approach), whereas FAMHE respectively takes 69 and 812 minutes when the same computations are collaboratively executed by 12 data providers. The centralized approach, contrarily to FAMHE, cannot distribute the workload among multiple DPs and suffers from a high overhead brought by centralized cryptographic operations. For example, in a centralized setting, a ciphertext is refreshed or bootstrapped in 26 seconds[32] for a security level of 108 bits, whereas the corresponding interactive protocol in FAMHE takes 0.6 seconds with a better security level of 128 bits. Blatt et al.[33] recently proposed two centralized HE-based approaches for GWAS. The authors rely either on a χ^2 test or on logistic regression. The former is optimized for performance by excluding the covariates, at the cost of lowering the obtained accuracy. The latter accepts a maximum of 3 covariates and can perform a limited number of iterations, due to the use of a centralized HE scheme with conservative cryptographic parameters. In both cases, their solutions scale linearly with the number of patients and variants considered, achieving estimated runtimes in our scenario of 50 minutes with χ^2 test and 16320 minutes with logistic regression. We note that FAMHE-FastGWAS performs the same operation but by including 13 covariates in 69 minutes. Moreover, FAMHE scales efficiently to large numbers of covariates and is not limited in the number of training iterations due to its efficient use of a multiparty HE-scheme. SMC approaches usually target settings with 2 to 4 parties due to the communication cost that becomes prohibitive for higher numbers of parties. FAMHE also works with 2 to 4 parties but is not specifically optimized for this scenario and its execution time would be in the same order of magnitude as secret-sharing-based solutions. However, unlike secret-sharing-based solutions, FAMHE efficiently scales to federated learning settings where many DPs keep their data locally (or in their own cloud). Furthermore, by designing an alternative MHE-friendly algorithm for the same task (e.g. FAMHE-FastGWAS), FAMHE can achieve faster execution times than SMC even in the setting with a small number of parties.

We summarize this comparison in Table 2. The two Decentralized approaches refer to cleartext adaptations of FAMHE's approaches. The results for Centralized, Non-Secure and Meta-analysis, Non-Secure are obtained through experiments with the PLINK software. The execution times for the Decentralized approaches are inferred from Meta-analysis, Non-Secure and their communication overheads are deduced from FAMHE's communication costs from which we removed the encryption overhead. We also relied on this encryption space overhead to estimate Centralized HE communication cost, whereas its execution time is deducted from our previous observation on the centralized bootstrapping. We estimated the performance of SMC by extrapolating the published results of Cho et al.[17]. For a fair comparison, we considered only the Phase 3 results, i.e., the association tests, for which Cho et al. computed the χ^2 statistics of the generalized Cochran-Armitage trend test corrected for covariates. We note that, while Cho et al. take a different approach to computing the association results than FAMHE, the overall workflow is similar and represents a suitable reference point for comparison. Since the prior work considered a three-party setting, we expect the real execution time of SMC to be higher in our setting with 12 DPs. For the communication cost of SMC, we combined the estimates of both initial data sharing and the main computation step (Phase 3) from the original publication and assumed linear scaling with the number of DPs. FAMHE measurements are obtained through our experiments.

	Centralized, Non-Secure	Meta-analysis, Non-Secure	Decentralized Non-secure	Decentralized, DiffP.	Centralized HE (FAMHE-approaches)	Centralized HE	SMC	FAMHE
Execution Time (min)	0.24	5	10	10	2228; 30781	50; 16320	320	69; 812
Communication cost (GB)	4	0.5	39; 171	39; 171	12	12	3144	122; 684

Supplementary Table 2. Quantitative comparison of existing approaches for biomedical FA. In distributed cases, the data are split among 12 data providers. The communication cost corresponds to the amount of data that one DP has to send during the entire process. When two values are provided, the left one corresponds to an approach equivalent to FastGWAS and the right one to GWAS.

Supplementary Note 6: Data Providers Independent Results

Supplementary Figure 4 shows the independent GWAS result obtained by each of the 12 data providers on its own subset of the data.

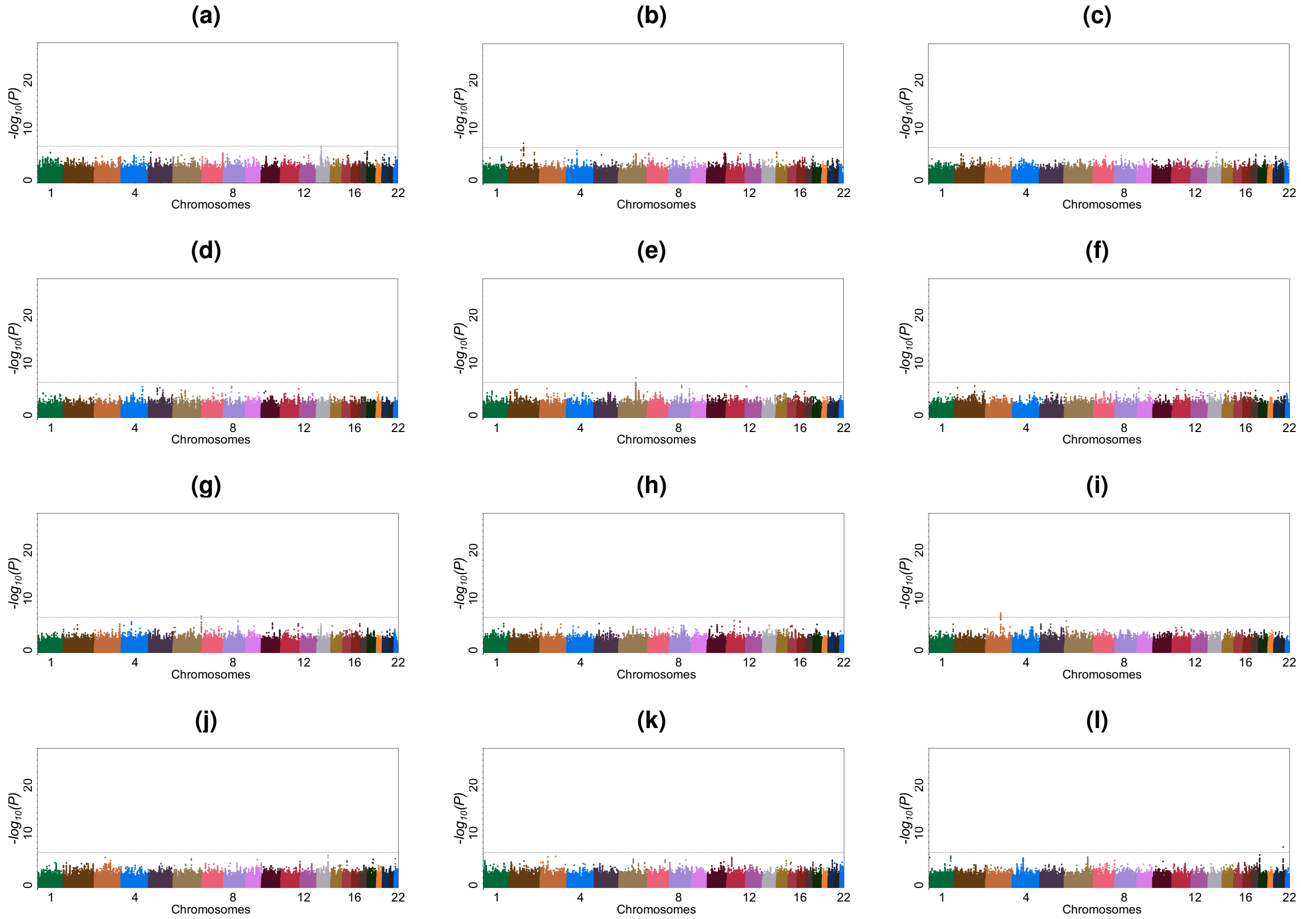

Supplementary Figure 4. Independent GWAS Results. Results obtained by each data provider using only its own subset of the data. The p-values shown are nominal values without multiple testing correction and are obtained using standard two-sided t-tests for testing whether the linear regression coefficient associated with a variant is non-zero.

Supplementary Note 7: Table of Symbols

Symbol	Description
FA	Federated Analytics
ML	Machine Learning
DP	Data Provider
DiffP	Differential Privacy
CA	Collective Aggregation
KS	Key Switching
MHE	Multiparty Homomorphic Encryption
SMC	Secure Multiparty Computation
$\mathbf{w}$	Model coefficients/weights
pk	Collective public key
N	Number of values encrypted in one ciphertext
SIMD	Single Instruction, Multiple Data
S	Number of data providers
t_j	Time when at least one event happened
d_j	Number of events at time t_j
n_j	The number of individuals known to have survived (or at risk)
$\hat{S}(t)$	Kaplan-Meier Estimator
T	Number of data points
p	Number of patients
f	Number of features
$\mathbf{X} \in \mathbb{R}^{p \times f}$	Covariates matrix
$\mathbf{y} \in \mathbb{R}^{p \times 1}$	phenotype or label
v	Number of variants
$\mathbf{u} \in \mathbb{R}^{p \times 1}$	Vector of 1 variant value for all patients
$\mathbf{V} \in \mathbb{R}^{p \times v}$	Variants matrix
p_{val}	P-value
pnorm	Cumulative distribution function (CDF) of the standard normal distribution
$\mathbf{w}[f+2]$	Weight corresponding to the variant
$\mathbf{y}'$	Prediction vector
mse	Mean Squared Error
p_i	DP_i's subset patients
DP_R	Root of the tree
$\text{E}()$	Encrypted value
GJ	Gauss-Jordan method
Q	Querier

Supplementary Table 3. Frequently Used Symbols and Notations.

References

1. Mouchet, C., Troncoso-pastoriza, J. R., Bossuat, J.-P. & Hubaux, J.-P. Multiparty Homomorphic Encryption from Ring-Learning-with-Errors. In *Tech. Report https://eprint.iacr.org/2020/304* (2019).
2. Fan, J. & Vercauteren, F. Somewhat Practical Fully Homomorphic Encryption. *IACR Cryptol. ePrint Arch.* (2012).
3. Cheon, J. H., Kim, A., Kim, M. & Song, Y. Homomorphic Encryption for Arithmetic of Approximate Numbers. In *ASIACRYPT* (2017).
4. Froelicher, D. *et al.* Scalable Privacy-Preserving Distributed Learning. *Proc. on Priv. Enhancing Technol. Symp.* (2021).
5. Lyubashevsky, V., Peikert, C. & Regev, O. On Ideal Lattices and Learning with Errors over Rings. In *EUROCRYPT* (2010).
6. All of Us Research Program, NIH. https://allofus.nih.gov/, (accessed: January 2021).
7. Genomics England. https://www.genomicsengland.co.uk/, (accessed: January 2021).
8. UK Biobank. https://www.ukbiobank.ac.uk/, (accessed: January 2021).
9. Sheller, M. J. *et al.* Federated Learning in Medicine: Facilitating Multi-institutional Collaborations without Sharing Patient Data. *Sci. reports* **10**, 1–12 (2020).
10. Nasirigerdeh, R. *et al.* sPLINK: A Federated, Privacy-Preserving Tool as a Robust Alternative to Meta-Analysis in Genome-Wide Association Studies. *BioRxiv* (2020).
11. Gaye, A. *et al.* DataSHIELD: Taking the Analysis to the Data, not the Data to the Analysis. *Int. journal epidemiology* **43**, 1929–1944 (2014).
12. Wang, Z. *et al.* Beyond Inferring Class Representatives: User-Level Privacy Leakage From Federated Learning. In *IEEE INFOCOM* (2019).
13. Melis, L., Song, C., De Cristofaro, E. & Shmatikov, V. Exploiting Unintended Feature Leakage in Collaborative Learning. In *IEEE Symposium on Security and Privacy (SP)*, 691–706 (2019).
14. Nasr, M., Shokri, R. & Houmansadr, A. Comprehensive Privacy Analysis of Deep Learning: Passive and Active White-box Inference Attacks against Centralized and Federated Learning. In *IEEE Symposium on Security and Privacy (SP)* (2019).
15. Plink Software. https://www.cog-genomics.org/plink/, (accessed: November 2020).
16. Jagadeesh, K. A., Wu, D. J., Birgmeier, J. A., Boneh, D. & Bejerano, G. Deriving Genomic Diagnoses without Revealing Patient Genomes. *Science* **357**, 692–695 (2017).
17. Cho, H., Wu, D. J. & Berger, B. Secure Genome-Wide Association Analysis using Multiparty Computation. *Nat. biotechnology* **36**, 547–551 (2018).
18. Froelicher, D. *et al.* Unlynx: A Decentralized System for Privacy-Conscious Data Sharing. *PETS* (2017).
19. Raisaro, J. L. *et al.* Medco: Enabling Secure and Privacy-Preserving Exploration of Distributed Clinical and Genomic Data. *IEEE/ACM Transactions on Comput. Biol. Bioinforma.* (2018).
20. Froelicher, D., Troncoso-Pastoriza, J. R., Sousa, J. S. & Hubaux, J. Drynx: Decentralized, Secure, Verifiable System for Statistical Queries and Machine Learning on Distributed Datasets. *IEEE TIFS* DOI: 10.1109/TIFS.2020.2976612 (2020).
21. Lu, Y., Zhou, T., Tian, Y., Zhu, S. & Li, J. Web-Based Privacy-Preserving Multicenter Medical Data Analysis Tools Via Threshold Homomorphic Encryption: Design and Development Study. *J. medical Internet research* **22**, e22555 (2020).
22. Bonomi, L., Jiang, X. & Ohno-Machado, L. Protecting Patient Privacy in Survival Analyses. *J. Am. Med. Informatics Assoc.* **27**, 366–375 (2020).
23. Li, W. *et al.* Privacy-Preserving Federated Brain Tumour Segmentation. In *MLMI* (2019).
24. Simmons, S., Sahinalp, C. & Berger, B. Enabling privacy-preserving gwass in heterogeneous human populations. *Cell systems* **3**, 54–61 (2016).
25. Jayaraman, B. & Evans, D. Evaluating Differentially Private Machine Learning in Practice. In *USENIX Security* (2019).
26. Moncada-Torres, A., Martin, F., Sieswerda, M., van Soest, J. & Geleijnse, G. VANTAGE6: an open source priVAcy preserviNg federaTed leArninG infrastructurE for Secure Insight eXchange. In *AMIA Annual Symposium Proceedings*, 870–877 (2020).
27. Data Sharing Network (SHRINE). https://www.i2b2.org/work/shrine.html, (accessed: January 2021).
28. Warnat-Herresthal, S. *et al.* Swarm Learning as a Privacy-preserving Machine Learning Approach for Disease Classification. *Nature* **594**, 265–270 (2021).

29. Sherman, J. & Morrison, W. J. Adjustment of an Inverse Matrix Corresponding to a Change in One Element of a Given Matrix. *The Annals Math. Stat.* **21**, 124–127 (1950).

30. WITDOM: empoWering prIvacy and securiTy in non-trusteD envirOnMents. https://cordis.europa.eu/project/id/644371/results, (accessed: January 2021).

31. McLaren, P. J. *et al.* Polymorphisms of Large Effect Explain the Majority of the Host Genetic Contribution to Variation of HIV-1 Virus Load. *Proc. Natl. Acad. Sci.* **112**, 14658–14663 (2015).

32. Han, K. & Ki, D. Better bootstrapping for approximate homomorphic encryption. In *CT-RSA* (2020).

33. Blatt, M., Gusev, A., Polyakov, Y. & Goldwasser, S. Secure large-scale genome-wide association studies using homomorphic encryption. *Proc. Natl. Acad. Sci.* **117**, 11608–11613 (2020).
