# Supplementary material for: Truly privacy-preserving federated analytics for precision medicine with multiparty homomorphic encryption
Source: Nat Commun. 2021 Oct 11;12:5910. doi: 10.1038/s41467-021-25972-y (PMC8505638; doi:10.1038/s41467-021-25972-y)
Supplement: Supplementary file 2 — Reporting Summary [file 41467_2021_25972_MOESM2_ESM.pdf]

nature portfolio

Corresponding author(s): David Froelicher

Last updated by author(s): Aug 9, 2021

Reporting Summary

Nature Portfolio wishes to improve the reproducibility of the work that we publish. This form provides structure for consistency and transparency in reporting. For further information on Nature Portfolio policies, see our Editorial Policies and the Editorial Policy Checklist.

Statistics

For all statistical analyses, confirm that the following items are present in the figure legend, table legend, main text, or Methods section.

n/a	Confirmed	
☐	☒	The exact sample size (n) for each experimental group/condition, given as a discrete number and unit of measurement
☐	☒	A statement on whether measurements were taken from distinct samples or whether the same sample was measured repeatedly
☐	☒	The statistical test(s) used AND whether they are one- or two-sided<br>*Only common tests should be described solely by name; describe more complex techniques in the Methods section.*
☒	☐	A description of all covariates tested
☒	☐	A description of any assumptions or corrections, such as tests of normality and adjustment for multiple comparisons
☒	☐	A full description of the statistical parameters including central tendency (e.g. means) or other basic estimates (e.g. regression coefficient) AND variation (e.g. standard deviation) or associated estimates of uncertainty (e.g. confidence intervals)
☐	☒	For null hypothesis testing, the test statistic (e.g. F, t, r) with confidence intervals, effect sizes, degrees of freedom and P value noted<br>*Give P values as exact values whenever suitable.*
☒	☐	For Bayesian analysis, information on the choice of priors and Markov chain Monte Carlo settings
☒	☐	For hierarchical and complex designs, identification of the appropriate level for tests and full reporting of outcomes
☒	☐	Estimates of effect sizes (e.g. Cohen's d, Pearson's r), indicating how they were calculated

Our web collection on statistics for biologists contains articles on many of the points above.

Software and code

Policy information about availability of computer code

Data collection

No specific software is used in order to collect the data.

Data analysis

We relied on the open-source softwares PLINK1.9 and PLINK2.0 (https://www.cog-genomics.org/plink/) for the execution of a GWAS in cleartext.
We relied on the R language to implement the Kaplan Meier Survival Analysis algorithm in a centralized manner and on the GoLang language to implement the decentralized computation of a Kaplan Meier Survival Analysis. We also relied on GoLang to implement the decentralized computation of a GWAS. For both implementations, we built on the public cryptographic library Lattigo (https://github.com/ldsec/lattigo) and on the public Onet library (https://github.com/dedis/onet) for communication among the different parties.
To implement the stochastic gradient descent, we relied on the privacy-preserving decentralized implementation with multiparty homomorphic encryption by Froelicher et al. (https://petsymposium.org/2021/files/papers/issue2/popets-2021-0030.pdf).
To implement the inverse of the covariance matrix, we relied on the Gauss-Jordan (GJ) method.
To implement the efficient integration of a new variable in a covariance matrix, we relied on the Shermann-Morrison-formula-based method presented in the report on Cryptographic and Privacy-preserving primitives (page 52) of the WITDOM European project (http://www.witdom.eu/).

Our solution relies on open-source softwares and public libraries. Our code is currently not publicly available as it is part of a licensing effort in a startup. Upon request, we will provide binaries that, in combination with open-source resources, will enable verifiability and reproducibility of our experiments.

For manuscripts utilizing custom algorithms or software that are central to the research but not yet described in published literature, software must be made available to editors and reviewers. We strongly encourage code deposition in a community repository (e.g. GitHub). See the Nature Portfolio guidelines for submitting code & software for further information.

Data

Policy information about availability of data

All manuscripts must include a data availability statement. This statement should provide the following information, where applicable:

- Accession codes, unique identifiers, or web links for publicly available datasets
- A description of any restrictions on data availability
- For clinical datasets or third party data, please ensure that the statement adheres to our policy

We replicated two existing medical studies, i.e., Samstein et al. (https://pubmed.ncbi.nlm.nih.gov/30643254/) and McLaren et al. (https://pubmed.ncbi.nlm.nih.gov/26553974), and refer the reader to these works to obtain the related datasets.

Field-specific reporting

Please select the one below that is the best fit for your research. If you are not sure, read the appropriate sections before making your selection.

☒ Life sciences ☐ Behavioural & social sciences ☐ Ecological, evolutionary & environmental sciences

For a reference copy of the document with all sections, see nature.com/documents/nr-reporting-summary-flat.pdf

Life sciences study design

All studies must disclose on these points even when the disclosure is negative.

Sample size	For both computations (i.e., survival curves and GWAS) we relied on the total number of samples available in the original datasets. We reproduced both studies by using all data samples and by distributing evenly the samples among different number of nodes. We replicated the available data samples to obtain enough (i.e., at least 10x) data samples to show our solution's scaling.
Data exclusions	We did not exclude any data from the obtained original datasets.
Replication	Our solutions are deterministic and give consistent results when ran on the same original data.
Randomization	In our experiments, the data are randomly distributed among the data providers. Each experiment is repeated 10x to ensure that the data distribution does not affect the final results.
Blinding	Blinding is not relevant to our study as we reproduced existing studies.

Reporting for specific materials, systems and methods

We require information from authors about some types of materials, experimental systems and methods used in many studies. Here, indicate whether each material, system or method listed is relevant to your study. If you are not sure if a list item applies to your research, read the appropriate section before selecting a response.

Materials & experimental systems

n/a	Involved in the study	
☒	☐	Antibodies
☒	☐	Eukaryotic cell lines
☒	☐	Palaeontology and archaeology
☒	☐	Animals and other organisms
☒	☐	Human research participants
☒	☐	Clinical data
☒	☐	Dual use research of concern

Methods

n/a	Involved in the study	
☒	☐	ChIP-seq
☒	☐	Flow cytometry
☒	☐	MRI-based neuroimaging
